# Helminth Antigens Modulate Virus-Induced Activation of CD154 (CD40L) Expression on T Cells in *Onchocerca volvulus*-Infected Individuals

**DOI:** 10.3390/pathogens15010093

**Published:** 2026-01-15

**Authors:** Brice Armel Nembot Fogang, Kathrin Arndts, Tomabu Adjobimey, Michael Owusu, Vera Serwaa Opoku, Derrick Adu Mensah, John Boateng, Jubin Osei-Mensah, Julia Meyer, Ute Klarmann-Schulz, Sacha Horn, Inge Kroidl, Alexander Y. Debrah, Achim Hoerauf, Manuel Ritter, Linda B. Debrah

**Affiliations:** 1 Department of Clinical Microbiology, School of Medical Sciences, Kwame Nkrumah University of Science and Technology, Kumasi PMB UPO, Ghana; nembotfogang@gmail.com (B.A.N.F.); jhnboat2008@gmail.com (J.B.); 2 Instiute for Medical Microbiology, Immunology and Parasitology (IMMIP), University Hosital Bonn, University of Bonn, 53127 Bonn, Germany; kathrin.arndts@ukbonn.de (K.A.); tomabuadjobi@hotmail.com (T.A.); julia.meyer@ukbonn.de (J.M.); ute.klarmann-schulz@uni-bonn.de (U.K.-S.); hoerauf@uni-bonn.de (A.H.); 3 Kumasi Centre for Collaborative Research in Tropical Medicine (KCCR), Kwame Nkrumah University of Science and Technology, Kumasi PMB UPO, Ghana; michaelowusu80@gmail.com (M.O.); vopoku88@gmail.com (V.S.O.); jubinom@yahoo.com (J.O.-M.); yadebrah@yahoo.com (A.Y.D.); 4 German West African Centre for Global Health and Pandemic Prevention (G-WAC), Partner Site Kumasi, Kumasi PMB UPO, Ghana; 5 German West African Centre for Global Health and Pandemic Prevention (G-WAC), Partner Site Bonn, 53127 Bonn, Germany; 6 Faculté des Sciences et Techniques (FAST), Université d’Abomey Calavi, Abomey Calavi 05 BP 1604, Benin; 7 Department of Medical Diagnostics, Kwame Nkrumah University of Science and Technology, Kumasi PMB UPO, Ghana; 8 Department of Theoretical and Applied Biology, Kwame Nkrumah University of Science and Technology, Kumasi PMB UPO, Ghana; derrickadumensah@yahoo.com; 9 Department of Public Health Education, Akenten Appiah-Menka University of Skills Training and Entrepreneurial Development, Kumasi P.O. Box 1277, Ghana; 10 Department of Medical Laboratory Technology, Royal Ann College of Health, Kumasi P.O. Box KS 6253, Ghana; 11 Department of Pathobiology, School of Veterinary Medicine, Kwame Nkrumah University of Science and Technology, Kumasi PMB UPO, Ghana; 12 German Center for Infection Research (DZIF), Partner Site Bonn-Cologne, 53127 Bonn, Germany; 13 Institute of Infectious Diseases and Tropical Medicine, LMU University Hospital, 80802 Munich, Germanyinge.kroidl@med.uni-muenchen.de (I.K.); 14 German Centre for Infectious Disease Research (DZIF), Partner Site Munich, 80802 Munich, Germany

**Keywords:** helminth, SARS-CoV-2, virus, co-infection, immune modulation, CD4^+^ T cell, CD8^+^ T cell, CD154 (CD40L)^+^ T cell

## Abstract

Background: The interaction between helminth and viral infections has important implications for understanding viral disease outcomes and vaccine efficacy in helminth-endemic regions. We previously demonstrated that helminth seropositivity is associated with reduced Th1/Th17 cytokine levels and reduced COVID-19 severity; however, the underlying immunological mechanisms remain unclear. This study further investigated these mechanisms by assessing how helminth antigens influence SARS-CoV-2-induced T-cell responses in individuals infected with filarial parasites *in vitro*. Methods: Peripheral blood mononuclear cells (PBMCs) from 43 participants, including *Onchocerca volvulus*-infected individuals, filarial lymphedema patients, and non-endemic controls, were stimulated *in vitro* with SARS-CoV-2 peptides and *Ascaris lumbricoides* antigens. Results: Fluorescence-activated cell sorting analysis showed a significant reduction in SARS-CoV-2-induced CD154 expression on CD4^+^ T cells but an increase on CD8^+^ T cells in *O. volvulus*-infected participants (*p* < 0.0001). *A. lumbricoides* antigens alone did not induce significant T-cell activation in *O. volvulus*-infected individuals. However, SARS-CoV-2 peptides strongly activated CD4^+^CD154^+^ T cells response (*p* = 0.0074), but co-stimulation with *A. lumbricoides* antigens markedly reduced CD3^+^ and CD4^+^CD154^+^ T-cell expression frequencies (*p* = 0.0329 and *p* = 0.0452). *A. lumbricoides*-specific IgG correlated inversely with SARS-CoV-2-induced CD4^+^CD154^+^ expression (r = −0.6025, *p* = 0.0049), whereas SARS-CoV-2-specific IgG was positively associated with CD4^+^CD154^+^ and CD8^+^CD154^+^ T-cell responses (β = 0.532, *p* = 0.016 and β = 0.509, *p* = 0.022). Conclusion: These findings demonstrate that helminth antigens modulate functional SARS-CoV-2-induced T-cell responses, offering a potential mechanism through which helminth co-infections shape antiviral immunity, vaccine efficacy, and clinical disease outcomes.

## 1. Introduction

The coronavirus disease 2019 (COVID-19), caused by the severe acute respiratory syndrome coronavirus 2 (SARS-CoV-2), emerged in late 2019 in Wuhan, China, and rapidly escalated into a global pandemic. Initially characterised by pneumonia-like symptoms, SARS-CoV-2 was officially identified in January 2020. This led to the unprecedented global health crisis, with the subsequent declaration of a Public Health Emergency of International Concern by the World Health Organisation (WHO) in the same month [1,2]. Over time, the virus evolved, giving rise to multiple variants of concern (VOCs), each associated with varying degrees of transmissibility and virulence. Notable strains include the Alpha (α), Beta (β), gamma (γ), Delta (δ) and the Omicron (σ) variant, which emerged in late 2021, significantly altering the global epidemiological landscape [2,3].

COVID-19 presents with a wide spectrum of clinical manifestations, ranging from asymptomatic cases to severe, life-threatening conditions. Asymptomatic individuals harbour the virus without evident symptoms, while severe cases are characterised by acute respiratory distress syndrome (ARDS), multi-organ failure, and hyperinflammatory responses often driven by a dysregulated immune system [4]. Severe cases are linked to a hyperactive immune response, including a cytokine storm marked by elevated levels of IL-6, IL-1β, and TNF-α, which cause tissue damage and exacerbate disease severity [5].

Globally, as of March 2024, COVID-19 has caused over 700 million confirmed cases and more than 6.8 million deaths [6]. While many international organisations, including the WHO, predicted catastrophic outcomes for Africa due to its fragile healthcare system and infrastructure, the continent performed unexpectedly well, with significantly lower mortality rates compared to Europe and other developed regions [7]. Although various factors, including a younger population, robust community-based health systems, and prior exposure to infectious agents, have been posited to explain Africa’s relatively favourable outcomes [8], immunomodulation by chronic parasitic infections, such as helminths (*Ascaris lumbricoides*, *Ancylostoma duodenale*, *Onchocerca volvulus*, *Wuchereria bancrofti*), has been suggested as a potential contributor to this phenomenon [9,10,11,12,13,14].

Helminths are parasitic worms that chronically infect billions of people in tropical and subtropical regions. They are transmitted through ingestion of contaminated food or water, skin penetration by infective larvae from soil or water, or via vectors or intermediate hosts, depending on the species. They are renowned for their ability to modulate the host’s immune system [15]. They modulate disease dynamics and vaccination success in the era of emerging infectious diseases by promoting a regulatory and anti-inflammatory response, often skewing the immune system toward a Th2 and regulatory-dominant profile, which may dampen hyperinflammatory responses associated with severe respiratory virus disease outcomes, including COVID-19 [13,16,17]. Evidence from other diseases has illustrated the profound impact of helminths on co-infections on disease outcome. For instance, helminth infections modulate the immune response and reduce the severity of allergic airway inflammation in tuberculosis, Respiratory syncytial Virus (RSV), COVID-19 and influenza [18,19,20]. Co-infection is a critical determinant of human health, particularly in helminth-endemic regions where chronic parasitic infections commonly coexist with viral pathogens. Such immune interactions shape pathogen-specific responses and can influence disease susceptibility and immune activation. Coinfection is particularly important in human health, as it directly tests how helminth antigens modify immune responses to viral antigens at the cellular level, a process directly addressed in the present study [21].

Upon viral entry into the host, the immune system initiates a cascade of events to neutralise the pathogen. Antigen-presenting cells (APCs) such as dendritic cells capture viral antigens and present them via MHC class II molecules to CD4^+^ T cells, triggering adaptive immunity [22]. Activated T cells, including CD154^+^ (CD40L), IFN-γ^+^, and TNF-α^+^ subsets, play pivotal roles in orchestrating antiviral responses. CD154^+^ T cells facilitate APC activation, IFN-γ^+^ T cells enhance antiviral defences by activating macrophages and NK cells, and TNF-α^+^ T cells mediate inflammatory responses crucial for pathogen clearance [23].

The expression of these T-cell markers occurs at distinct time points post-immune activation. CD154^+^ expression peaks within hours of antigen presentation, while IFN-γ^+^ and TNF-α^+^ T cells become more prominent within 24–48 h of stimulation [24,25,26], reflecting their critical roles in immune responses [27,28,29]. Recent evidence highlights the importance of these markers in controlling COVID-19 severity, as robust T-cell responses and hyperimmune activation correlate with severe disease outcomes [30,31].

Building on these insights, the present study aims to investigate the *in vitro* effect of helminth exposure on the expression of viral reactive T cells in lymphatic filariasis lymphedema participants from the Upper East Region and *O. volvulus*-infected participants from the Western North region of Ghana. By examining the *in vitro* effect of *A. lumbricoides* Ag on SARS-CoV-2-induced CD8^+^CD154^+^, CD4^+^CD154^+^, CD154^+^IFN-γ^+^, and CD154^+^TNF-α^+^ T lymphocytes expression, as well as their expression in active filarial infection of *O. volvulus* versus cleared filarial infection with pathology, we seek to elucidate the immunomodulatory influence of helminths on viral cellular immunity (T-cell responses). Our findings will have significant implications for understanding the interplay between chronic parasitic infections and viral immunity.

## 2. Materials and Methods

### 2.1. Study Design and Participant Recruitment

The study was conducted using 43 PBMC samples from participants living in the Western North (20), Upper East (17), and the Ashanti (6) Regions of Ghana. These samples were collected during the third wave of the pandemic from November 2021 to mid-2022 as part of a larger project studying filarial lymphedema in the Upper East Region, an endemic region for lymphatic filariasis (LF), and onchocerciasis in the Western North Region. These diseases are both debilitating parasitic diseases known to modulate the immune system. These regions were cold spots of COVID-19 during the peak of the pandemic. The Ashanti Region is a non-endemic region for these chronic infections; however, other Neglected Tropical Diseases (NTDs), such as ascariasis and hookworm infections, exist in this region with a lower prevalence. This low-helminth-endemic region was one of the COVID-19 hotspots during the pandemic in Ghana. The samples used included PBMCs from both unvaccinated and vaccinated individuals for SARS-CoV-2. The vaccinated group had received mRNA or viral vector vaccines. The unvaccinated group included both seropositive and seronegative individuals for SARS-CoV-2. Reflecting the region’s demographics, individuals of different age groups and genders were included in the study.

The recruitment was conducted by teams from the Kumasi Centre for Collaborative Research in Tropical Medicine (KCCR) in Ghana and the Institute of Medical Microbiology, Immunology and Parasitology (IMMIP) in Bonn, Germany, as part of a larger project, as previously described [32,33]. All personnel involved in sample collection were thoroughly trained and provided with standardised protocols. The demographic and clinical data of participants were collected using predefined questionnaires. Blood samples from consenting participants were collected in EDTA tubes under aseptic conditions and subsequently processed for PBMCs by density gradient centrifugation. The PBMCs were then aliquoted into tubes and stored in liquid nitrogen in Ghana before being shipped in liquid nitrogen to the Institute for Medical Microbiology, Immunology and Parasitology (IMMIP) of the University Hospital Bonn (UKB) for further analysis.

### 2.2. Onchocerca volvulus and Wuchereria bancrofti Assessment and Filarial Lymphedema (LE) Staging

For assessment of *O. volvulus* (Western North Region), participants were first palpated for the presence of onchocercomata (nodules). Detection of *O. volvulus* microfilaria from nodule-positive participants was carried out using the microscopy-based skin snip method [34]. A small piece of skin was aseptically snipped from the right and left iliac crests of each participant and immediately placed in 1 mL of normal saline in a 24-well microtitre plate. The samples were incubated overnight to allow any microfilariae to emerge. The saline solution was then examined under an inverted microscope for the presence of microfilaria. The remaining skin samples were preserved in 100% isopropanol for long-term storage and further analyses.

Participants with LF lymphedema (LE) involving at least one leg, graded 1–6, were also included in this study. LE was staged using Dreyer’s lymphedema staging system, which categorises the condition from stage 0 to stage 7 for each leg. Stage 0 indicates no abnormality; stage 1 is characterised by reversible swelling that resolves overnight; stage 2 involves non-reversible swelling; stage 3 is defined by shallow skin folds visible when the ankle is straightened; stage 4 is marked by skin knobs; stage 5 by deep skin folds, where the fold base is only visible when manually separated; stage 6 by “mossy lesions” representing warty epidermal changes; and stage 7 by severe disability, in which individuals are unable to care for themselves or perform daily activities [33].

To exclude ongoing LF infection among LE participants, the circulating filarial antigen (CFA) test was carried out using the Alere Filariasis Test Strip (FTS, Abbott Laboratories, Chicago, IL, USA). For this test, 75 µL of capillary finger-prick blood was applied to the sample pad, allowed to migrate, and read after 10 min. The presence of both the test and control bands was interpreted as a positive result, indicating CFA positivity.

### 2.3. Measurement of SARS-CoV-2-Specific Antibodies

Detection of antibodies directed against SARS-CoV-2 was carried out at the IMMIP, University Hospital Bonn, Germany. Plasma samples were tested for virus-specific IgA and IgG using the Euroimmun ELISA kits (Euroimmun, Lübeck, Germany). The ELISA wells were pre-coated with the S1 subunit of the SARS-CoV-2 spike protein. The assays were automated using the Euroimmun Analyser I (Euroimmun, Lübeck, Germany), strictly adhering to the manufacturer’s protocol [35]. Optical density readings were obtained at 450 nm, and the corresponding antibody titers were obtained.

### 2.4. Measurement of A. lumbricoides-Specific Antibodies

Plasma samples were also screened for IgG antibodies specific to *A. lumbricoides* antigens. Quantification was performed using the diagnosis-related group (DRG) *A. lumbricoides* IgG ELISA kit (DRG Instruments GmbH, Marburg, Germany). Assays were conducted manually, following the procedure described in the kit insert. Absorbance values were read at 450 nm with reference at 620 nm. Absorbance values were converted into DRG Units (DU) or Index according to the manufacturer’s instructions using the formula: (sample absorbance × 10)/cut-off. Results were interpreted following the manufacturer’s criteria, with values > 11 DU considered positive, 9–11 DU considered equivocal, and <9 DU considered negative.

### 2.5. Antigen Preparation

Soluble extracts from adult *A. lumbricoides* worms were prepared as previously described [31]. Frozen adult worms were thawed and placed in a Petri dish containing sterile phosphate-buffered saline (PBS). After several washes in PBS, the worms were transferred to a glass mortar (VWR, Langenfeld, Germany), where 5 mL of RPMI medium was added, and the worms were crushed to achieve a homogeneous solution. The resulting extracts were then centrifuged at 300× *g* for 10 min at 4 °C to remove insoluble material. The supernatants were collected into a new tube, and protein concentrations were determined using the Pierce Coomassie Plus (Bradford) Assay Kit (ThermoFisher Scientific, San Diego, CA, USA) as per the manufacturer’s protocol. The aliquots were stored at −80 °C until use. The optimal concentration for cell stimulation was determined via a titration assay, and endotoxin levels were measured using the Pierce Limulus Amoebocyte Lysate (LAL) Chromogenic Quantification Kit (ThermoFisher Scientific). The endotoxin levels were consistently below the detection limit of 0.1 EU/mL.

### 2.6. PBMC Isolation and Cryopreservation

Peripheral blood mononuclear cells (PBMCs) were isolated from 7 mL of whole blood using Leucosep tubes pre-filled with 3 mL of gradient separation medium. The blood was layered onto the barrier to prevent mixing with the gradient medium and centrifuged at 1000× *g* for 10 min at room temperature (acceleration 9, deceleration 0). Following centrifugation, PBMCs were harvested from the plasma-gradient interface using a Pasteur pipette, washed twice with Roswell Park Memorial Institute (RPMI) medium supplemented with 10% foetal bovine serum (FBS) at 1500 rpm for 10 min, and resuspended in 800 μL RPMI + 10% FBS. For cryopreservation, cells were mixed 1:1 with freezing medium (80% FBS, 20% Dimethyl Sulfoxide (DMSO)), aliquoted, and stored overnight in the gas phase of liquid nitrogen before transferring to liquid nitrogen for long-term storage.

### 2.7. Cell Treatment

A day before the experimental procedure, cells stored in liquid nitrogen were thawed as previously described [36]. Thawed cells were washed twice with 10 mL RPMI medium for 8 min at 1300 rpm. Recovery of cryopreserved cells was done by culturing them in 2 mL of RPMI supplemented with 10% FBS medium at 37 °C under 5% CO_2_ for overnight incubation. After cell recovery, they were quantified, and a viability count was performed using the trypan blue exclusion assay method [37]. Samples with a minimum viability count of 80% were included for the experimental assay.

### 2.8. Stimulation and Cell Culture

PBMCs stimulation for subsequent analysis of SARS-CoV-2-reactive T-lymphocytes was performed using the commercially available SARS-CoV-2 Select T-Cell Analysis Kit (PBMC) by Miltenyi Biotec, Bergisch Gladbach, Germany, following the manufacturer’s instructions [38]. SARS-CoV-2 peptide reference B142719 was used for stimulation. About 7 × 10^5^ viable cells per well were plated in a 96-well culture plate. The plated cells were total PBMCs, and they were treated afterwards for T-cell analysis. Stimuli included SARS-CoV-2 peptides (1 μg/mL) and *A. lumbricoides* antigens (20 μg/mL). These stimuli were used due to the co-endemicity of *A. lumbricoides* and the SARS-CoV-2 infection in this region (Ghana). Mono- and co-stimulation assays involved SARS-CoV-2 peptides and *A. lumbricoides* Ag. Cell culture medium (RPMI) without any stimulation was used as a negative control. After 2 h of stimulation, Brefeldin A (1 μg/mL) by Miltenyi Biotec was added to each well, and the cell culture plate was further incubated for 4 h. Following this incubation period, the cells were harvested in 100 μL of PEB buffer by Miltenyi Biotec and fixed in 100 μL of Inside Fix for 20 min at room temperature (20–25 °C), before being briefly permeabilised with 100 μL of Inside Perm for intracellular staining.

### 2.9. FACS Staining and Gating Strategy

**Figure 1:**
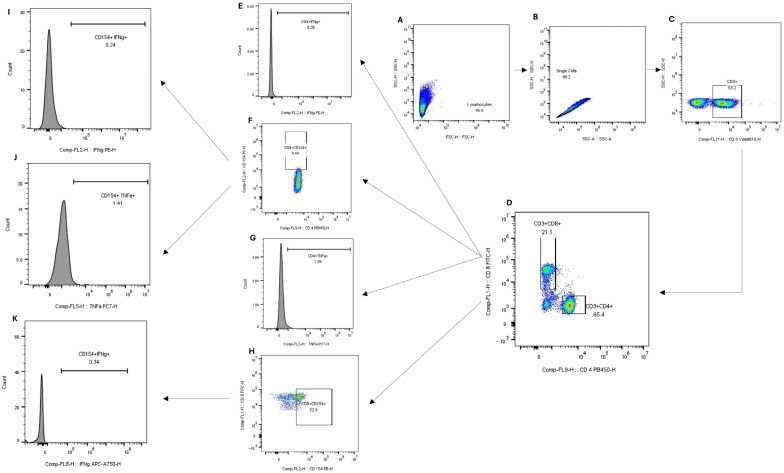
Gating strategy for CD4^+^ and CD8^+^ T cell activation markers. First, lymphocytes were identified by gating side scatter height (SSC-H) against forward scatter height (FSC-H) (**A**). From the lymphocyte population, single cells were gated using SSC-H versus side scatter area (SSC-A) (**B**). Subsequently, CD3^+^ T cells were gated using SSC-H against the fluorochrome corresponding to CD3 expression (**C**). From the CD3^+^ population, further gating was done to identify CD4^+^ and CD8^+^ T cell subsets (**D**). Within the CD4^+^ T cell population, IFN-γ^+^ T cells (PE-H) (**E**) and TNF-α^+^ T cells (PC7-H) (**G**) were gated. CD154^+^ cells were also identified from the CD4^+^ population using the appropriate fluorochrome (**F**). From the CD4^+^CD154^+^ population, further gating was performed to isolate dual-positive CD154^+^IFN-γ^+^ (**I**) and CD154^+^TNF-α^+^ cells (**J**). From the CD8^+^ T cell population, CD154^+^ cells were similarly gated (**H**), followed by the identification of IFN-γ^+^ cells within the CD8^+^CD154^+^ subset (**K**). Colour density represents the number of cells in each region of the plot, with warmer colours (yellow to red) indicating higher cell density and cooler colours (blue to green) indicating lower cell density.

Different fluorochromes were used across samples from the three regions: Western North (*O. volvulus*), Upper East (cleared filarial infection with lymphedema; LE LF), and Ashanti Regions (uninfected control). Staining for samples from the Western North Region targeted CD3^+^CD4^+^CD154^+^ and CD3^+^CD8^+^CD154^+^ T cells. Further staining was employed on samples from the Upper East Region to further analyse CD3^+^CD4^+^CD154^+^IFN-γ^+^ T cells and CD3^+^CD4^+^CD154^+^TNF-α^+^ T cells. All staining reagents were obtained from Miltenyi Biotec. The staining procedure was performed according to the manufacturer’s instructions [38]. Permeabilised cells were stained with 2 μL each of CD3 (clone: REA613), CD4 (clone: REA623), CD8 (clone: REA734), CD20 (clone: REA780), CD154 (clone: REA238), IFN-γ (clone: REA600), TNF-α (clone: REA656) and CD14 (clone: REA599), all from recombinant human IgG1 isotype antibodies, for 10 min at room temperature. Data was acquired from the cell suspension using CytoFLEX S Flow Cytometer manufactured by Beckman Coulter Biotechnology (Suzhou, China) Co., Ltd. Fluorescence compensation to correct spectral overlap was performed using staining controls (Single stains and unstained). To minimise subjectivity in identifying positive events, including CD154-positive events, fluorescence minus one (FMO) controls were used to define gating boundaries, and the same gating strategy was applied uniformly across all samples. FlowJo version 10 was used for cell gating. The gating strategy used to isolate different cell populations is illustrated in Figure 1. The frequency of cells was expressed as the percentage of parent cells (frequency of CD4^+^CD154^+^ T cells expressed as a percentage of CD4^+^ T cells, and frequency of CD8^+^CD154^+^ as a percentage of CD8.

### 2.10. Data Analysis

FlowJo version 10.10.0, IBM SPSS Statistics version 26, and Graphpad Prism version 10.2.0 were used to analyse the FACS data. Data were analysed for parametric distribution (Shapiro–Wilk test), and since they deviated from parametric assumptions, the Kruskal–Wallis test, followed by Dunn’s multiple comparisons test, was employed to compare differences in T cell expression across the various stimulation groups. The Mann–Whitney test was also used to compare T cell expression between two groups. A linear regression model was used to detect any association between clinical/serological status and SARS-CoV-2-induced T cell expression. More so, Spearman’s correlation test was used to find the correlation between *A. lumbricoides*-specific IgG expression and SARS-CoV-2-induced T cell response. Graphs were generated using GraphPad Prism version 10.2.0 (La Jolla, CA, USA). Statistical significance was considered at *p* < 0.05.

## 3. Results

### 3.1. Demographic and Clinical Characteristics of the Study Population

This study investigated the modulatory effects of helminth antigens on viral immunity using *in vitro* stimulation of PBMCs from participants across three regions of Ghana. The main cohort consisted of 20 *O. volvulus*-infected participants from the Western North Region, predominantly males (15/20, 75.0%) and mostly aged 25–44 years (13/20, 65.0%). Half of the participants were vaccinated against SARS-CoV-2 (10/20, 50.0%), while 15/20 (75.0%) were seropositive for SARS-CoV-2 antigens (Table 1). On average, participants presented with three palpable nodules per individual and an average of 56 MF/mg skin snip. The median levels of SARS-CoV-2 IgA and IgG in the cohort were 7.9ABU (IQR: 1.8–16.0) and 5.4 ABU (IQR: 1.3–8.3), respectively. The median expression of *A. lumbricoides*-specific IgG was 23.9 index (IQR: 16.3–28.5) (Table 1).

From the Upper East Region, 17 lymphedema participants with CFA-negative antigen test were included. The majority were female (15/17, 88.2%) and aged 45–64 years (10/17, 58.8%). Few were vaccinated (7/17, 41.2%), but most [16/17 (94.1%)] were seropositive for SARS-CoV-2. They were all CFA negative. However, all exhibited varying degrees of LF lymphedema (stages 1–6) in at least one leg (Table 1).

The Ashanti Region contributed six uninfected controls, comprising four men (4/6, 66.7%) and two women (2/6, 33.3%). Most were young adults, aged 25–44 years (5/6, 83.3%). These participants were CFA-negative and *A. lumbricoides* seronegative, with no history of chronic filarial infection. This non-endemic control group was fully vaccinated (6/6, 100.0%) and uniformly seropositive for SARS-CoV-2 (6/6, 100.0%), serving as the baseline for comparison with the filarial worm-infected/endemic group (Table 1).

**Figure 2:**
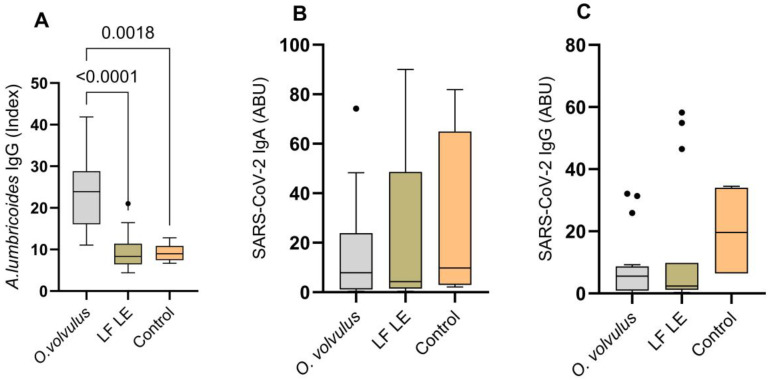
Expression of *A. lumbricoides* IgG (**A**), SARS-CoV-2-specific IgA (**B**) and SARS-CoV-2-specific IgG (**C**) among *O. volvulus*-infected participants, LF-LE (lymphatic filariasis-associated lymphedema) participants, and non-endemic controls. The control group were participants without *O. volvulus* infection or lymphatic filariasis. Boxplots show the median and interquartile range of antibody levels for each group. *A. lumbricoides* IgG levels are expressed as index values (DRG Units, DU), representing antibody titers in blood plasma calculated according to the manufacturer’s protocol. SARS-CoV-2 IgA and IgG levels are expressed as arbitrary binding units (ABU), reflecting plasma antibody titers determined following the manufacturer’s instructions. Group comparisons were performed using the Kruskal–Wallis test. Dots indicate outliers, defined as values that fall outside 1.5 times the interquartile range.

Although the entire study cohort was seropositive for *Ascaris lumbricoides* infection, *O. volvulus*-infected individuals showed a significant increase in *A. lumbricoides* IgG index (Figure 2A) compared to LF lymphedema and control participants. In addition, there was no significant difference in SARS-CoV-2 IgA and IgG between the *O. volvulus*-infected participants, LF lymphedema participants and the control group (Figure 2B,C).

### 3.2. Filarial Infection Suppresses SARS-CoV-2–Induced T Cell Activation

**Figure 3:**
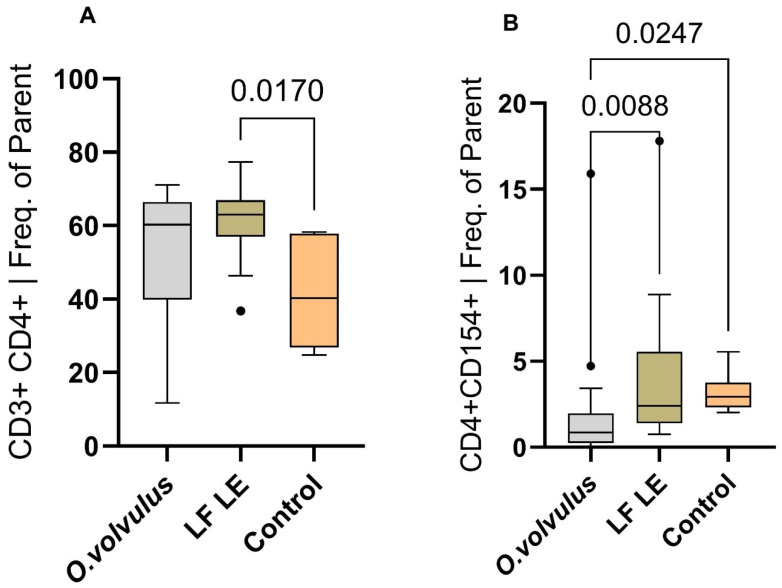
Effect of filarial worm infection on SARS-CoV-2–induced CD3^+^CD4^+^T cell (**A**) and CD4^+^CD154^+^T cell (**B**) activation. PBMCs were from *O. volvulus*-infected participants, LF LE participants, and a non-endemic control group. The control group were participants without *O. volvulus* infection or lymphatic filariasis. Approximately 7 × 10^5^ cells per donor were stimulated *in vitro* with SARS-CoV-2 peptides (1 µg/mL) for 6 h. Boxplots display the median and interquartile range of SARS-CoV-2–induced T cell responses in each group. Statistical analysis was performed using the Kruskal–Wallis test. “*O. volvulus*” denotes participants with active *O. volvulus* infection, while “LF LE” refers to individuals cleared of *Wuchereria bancrofti* filarial worms and who have lymphedema; “Control” refers to the non-endemic control group. Dots indicate outliers, defined as values that fall outside 1.5 times the interquartile range. The frequency of cells was expressed as the percentage of parent cells (Freq. of Parent).

PBMCs were stimulated with SARS-CoV-2 peptides, and the frequency of SARS-CoV-2–responsive T cells was compared between participants with active *O. volvulus* infection and those without the infection (LF LE and controls). Active filarial infection (*O. volvulus*) was significantly associated with reduced expression of SARS-CoV-2–induced CD4^+^CD154^+^ T cells compared to LF LE individuals and the control group (*p* = 0.0088 and *p* = 0.0247, respectively) (Figure 3). This suggests that active filarial infection may be necessary to induce a strong immune modulation and suppress virus-specific T cell activation.

### 3.3. A. lumbricoides Antigens Suppress SARS-CoV-2-Induced CD3^+^ and CD4^+^CD154^+^ T Cells in O. volvulus-Infected Individuals

**Figure 4:**
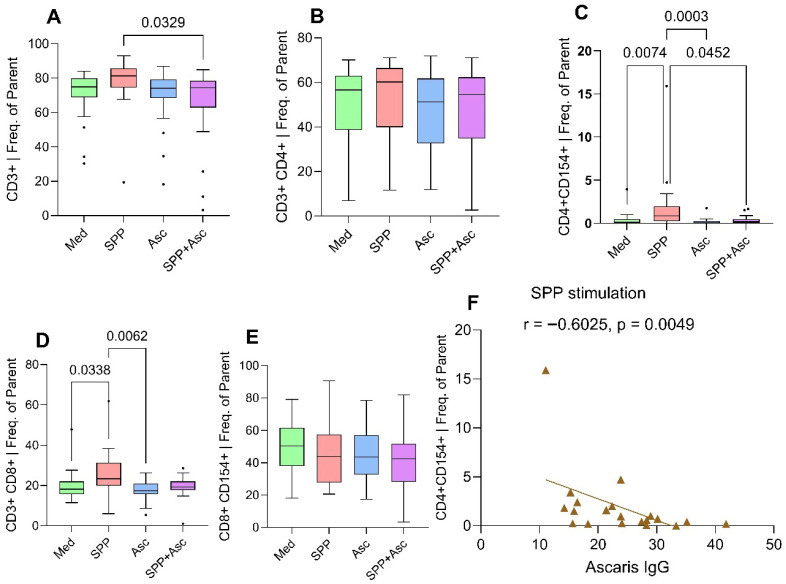
Effect of *A. lumbricoides* antigens on SARS-CoV-2-induced CD3^+^ T cell (**A**), CD3^+^CD4^+^ T cell (**B**), CD4^+^CD154^+^ T cell (**C**), CD3^+^CD8^+^ T cell (**D**), and CD8^+^CD154^+^ T cell (**E**) expression in *O. volvulus*-infected participants. The figure also illustrates the correlation between SARS-CoV-2-induced CD4+CD154+ T cells and *A. lumbricoides*-specific IgG (**F**): PBMCs were obtained from 20 *O. volvulus*-infected participants. Approximately 7 × 10^5^ cells from each donor were stimulated *in vitro* with *A. lumbricoides* antigen (20 µg/mL) and SARS-CoV-2 peptides (1 µg/mL) for a duration of 6 h. Boxplots represent the median and interquartile range expression of T cells for each stimulation group. Analysis was performed using the Kruskal–Wallis test followed by Dunn’s multiple comparison test. “Med” stands for control medium, “SPP” for SARS-CoV-2 peptide pool, “Asc” for *A. lumbricoides* antigens, “SPP + Asc” for SARS-CoV-2 peptide pool with *A. lumbricoides* antigens. From panel (**A**–**E**), dots indicate outliers, defined as values falling outside 1.5× the interquartile range. In panel (**F**), the triangles represent datapoints. The frequency of cells was expressed as the percentage of parent cells (Freq. of Parent).

**Figure A1:**
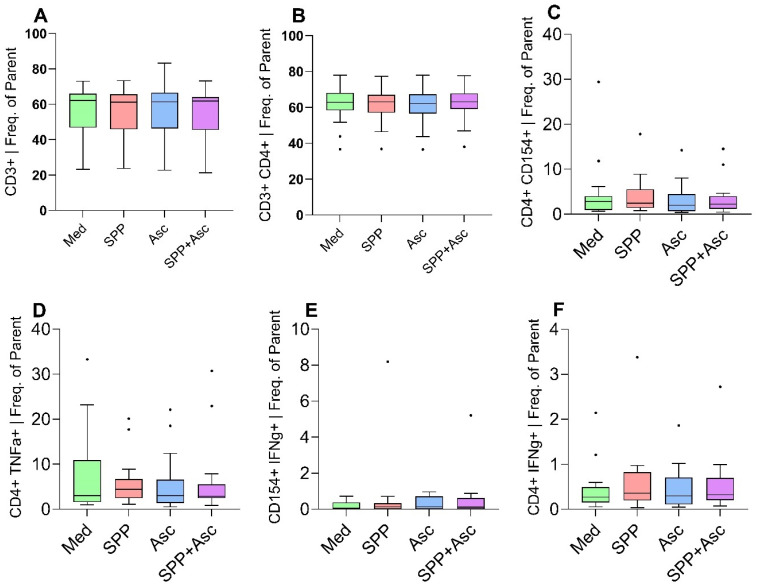
Expression of antigen-specific T cells in LF LE participants; The figure shows the effect of *A. lumbricoides* antigen stimulation on CD3^+^ T cells (**A**), CD3^+^CD4^+^ T cells (**B**), CD4^+^CD154^+^ T cells (**C**), CD4^+^TNF-α^+^ T cells (**D**), CD154^+^ IFN-γ^+^ T cells (**E**), and CD4^+^IFN-γ^+^ T cells (**F**). PBMCs were obtained from 17 LF LE donors. Approximately 7 × 10^5^ cells from each donor were stimulated *in vitro* with *A. lumbricoides* antigen (20 µg/mL) and SARS-CoV-2 peptides (1 µg/mL) for a duration of 6 h. Boxplots represent the median and interquartile range expression of T cells for each stimulation group. Comparison was performed using the Kruskal–Wallis test followed by Dunn’s multiple comparison test. “Med” stands for the control medium, “SPP” for SARS-CoV-2 peptide pool, “Asc” for *A. lumbricoides* antigens, “SPP + Asc” for *A. lumbricoides* with SARS-CoV-2 peptide pool combination. Dots indicate outliers.

Cells were stimulated with SARS-CoV-2 peptides (SPP) and *A. lumbricoides* antigens as a mono and co-stimulation. In *O. volvulus*-infected participants, *A. lumbricoides* antigens did not significantly elicit a T cell response. However, SARS-CoV-2 peptides robustly activated CD4^+^CD154^+^ and CD3^+^CD8^+^ T cells expression in relation to the control medium (*p* = 0.0074 and *p* = 0.0383, respectively). Co-stimulation with *A. lumbricoides* antigen resulted in a significant reduction in the expression of these cells (CD3^+^ and CD4^+^CD154^+^ T lymphocytes) compared to SARS-CoV-2 mono-stimulation (*p* = 0.0329 and *p* = 0.0452, respectively) (Figure 4A–E). The Spearman correlation between *A. lumbricoides* IgG and CD4^+^CD154^+^ T cells expression is illustrated in Figure 4F. *A. lumbricoides*-specific IgG showed an inverse correlation with SARS-CoV-2-induced CD4^+^CD154^+^ T cells response (r = −0.6025, *p* = 0.0049). Cells from individuals with cleared filarial infections (LF LE) did not show any significant difference in activated T cells or in cytokine-producing T cells upon stimulation with *A. lumbricoides* antigen and SPP (Figure A1). These findings suggest again that active infection is necessary to drive the modulation of the host immune system.

### 3.4. Downregulation of CD154 on CD4^+^ T Lymphocytes in O. volvulus-Infected Participants

**Figure 5:**
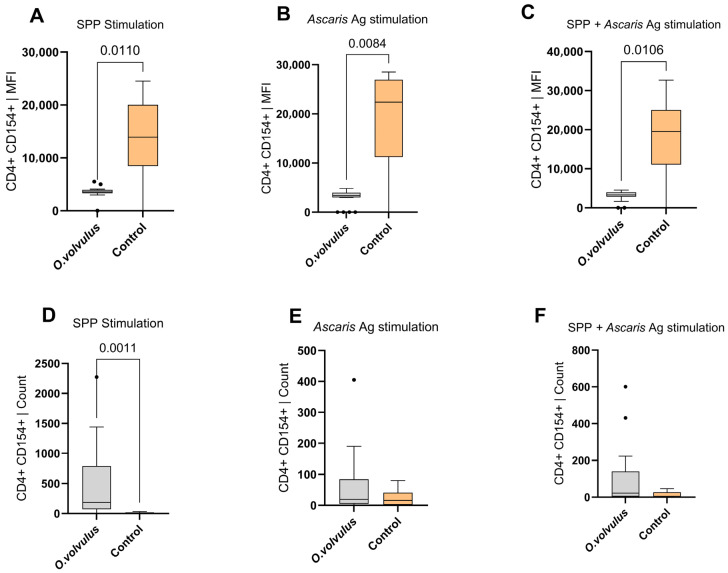
Comparison of CD4^+^CD154^+^ T lymphocyte mean fluorescence intensity (MFI) between *O. volvulus*-infected participants and non-endemic controls following stimulation with: SARS-CoV-2 peptides (SPP) (**A**), *A. lumbricoides* antigens (**B**), and combined SPP + *A. lumbricoides* antigens (**C**). Panels (**D**–**F**) show the corresponding cell counts under the same stimulation conditions: SPP (**D**), *A. lumbricoides* (**E**), and SPP + *A. lumbricoides* (**F**). The control group consisted of participants without *O. volvulus* infection or lymphatic filariasis. Approximately 7 × 10^5^ cells from each donor were stimulated *in vitro* with *A. lumbricoides* antigen (20 µg/mL) and SARS-CoV-2 peptides (1 µg/mL) for a duration of 6 h. Boxplots represent the median and interquartile range of T cell expression for each comparison group. Count refers to the number of individual cells that pass through the flow cytometer and are detected during a run. “SPP” stands for SARS-CoV-2 peptide pool, and MFI represent the mean fluorescence intensity. Comparison was performed using the Mann–Whitney test. Dots indicate outliers, defined as values that fall outside 1.5 times the interquartile range.

**Figure A2:**
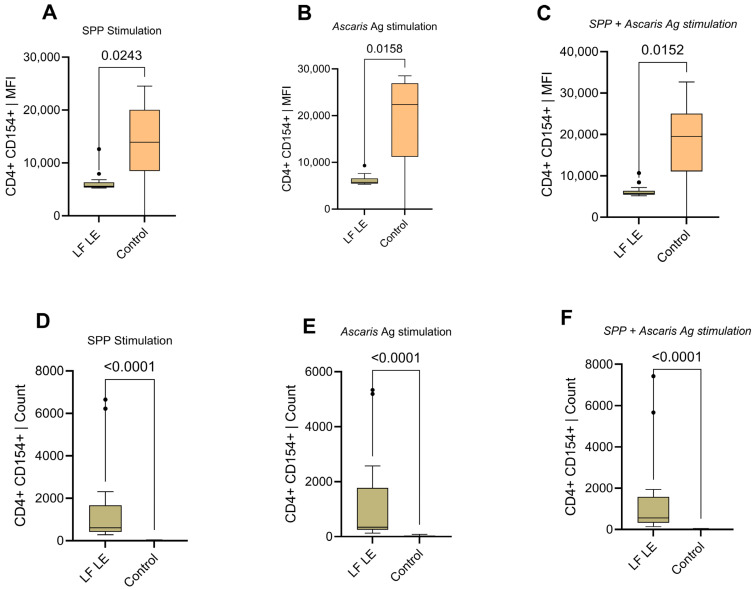
Comparison of CD4^+^CD154^+^ T cell expression between LF LE donors and non-endemic controls following stimulation with: SARS-CoV-2 peptides (SPP) (**A**), *A. lumbricoides* antigens (**B**), and combined SPP + *A. lumbricoides* antigens (**C**). Panels (**D**–**F**) show the corresponding cell counts under the same stimulation conditions: SPP (**D**), *A. lumbricoides* (**E**), and SPP + *A. lumbricoides* (**F**). Approximately 7 × 10^5^ cells from each donor were stimulated *in vitro* with *A. lumbricoides* antigen (20 µg/mL) and SARS-CoV-2 peptides (1 µg/mL) for a duration of 6 h. Boxplots represent the median and interquartile range of expression of T cells for each comparison group. Comparison was performed using the Mann–Whitney test. Dots indicate outliers.

The cell counts and mean fluorescence intensity (MFI) of CD4^+^CD154^+^ T cells were compared between *O. volvulus*-infected participants and non-endemic controls. The analysis revealed less CD154 (CD40L) expression on CD4^+^ T cells in *O. volvulus*-infected participants compared to controls (Figure 5A–C). Interestingly, this low expression occurred despite an increasing cell count (Figure 5D–F). Similarly, CD4^+^CD154^+^ T cell expression was lower in the LE LF participants despite an increasing cell count (Figure A2). The decrease in CD154 expression despite the increasing cell numbers could reflect lower activation of cells despite their increasing abundance.

### 3.5. Upregulation of CD8^+^CD154^+^ T Cells in the O. volvulus Patients Post-Stimulation

**Figure 6:**
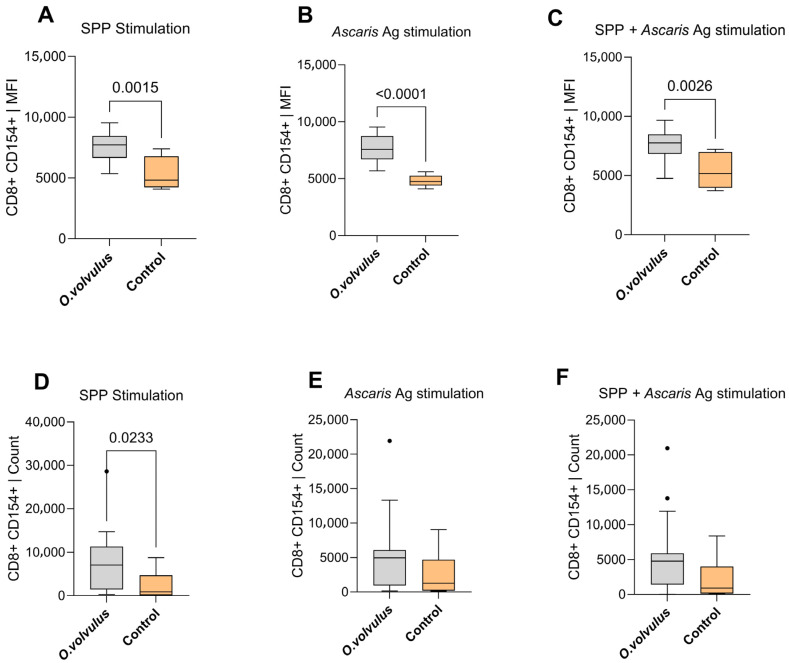
Comparison of CD8^+^CD154^+^ T lymphocyte mean fluorescence intensity (MFI) between *O. volvulus*-infected participants and non-endemic controls following stimulation with: SARS-CoV-2 peptides (SPP) (**A**), *A. lumbricoides* antigens (**B**), and combined SPP + *A. lumbricoides* antigens (**C**). Panels (**D**–**F**) show the corresponding cell counts under the same stimulation conditions: SPP (**D**), *A. lumbricoides* (**E**), and SPP + *A. lumbricoides* (**F**). The control group consisted of participants without *O. volvulus* infection or lymphatic filariasis. Approximately 7 × 10^5^ cells from each donor were stimulated *in vitro* with *A. lumbricoides* antigen (20 µg/mL) and SARS-CoV-2 peptides (1 µg/mL) for a duration of 6 h. Boxplots represent the median and interquartile range of T cell expression for each comparison group. Count refers to the number of individual cells that pass through the flow cytometer and are detected during a run. “SPP” stands for SARS-CoV-2 peptide pool, and MFI represent the mean fluorescence intensity. Comparison was performed using the Mann–Whitney test. Dots indicate outliers, defined as values that fall outside 1.5 times the interquartile range.

Interestingly, while a significantly lower expression of CD154 on CD4^+^ T cells was observed in *O. volvulus*-infected participants (Figure 5A–C), the expression of CD154 on CD8^+^ T cells was notably higher in the same participants (Figure 6A–C). This higher expression was preceded by a higher cell count in this group of participants (Figure 6D–F). This pattern suggests a progressive accumulation of CD8^+^CD154^+^ cells with enhanced activation status.

### 3.6. A. lumbricoides IgG is Associated with Reduced SARS-CoV-2 Antigen-Specific T Cell Activation in O. volvulus-Infected Participants

**Figure A3:**
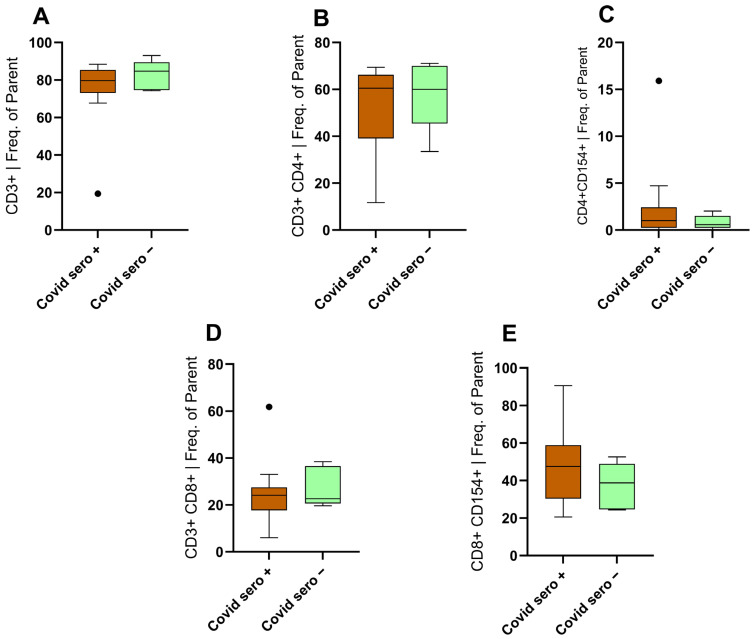
Comparison of SARS-CoV-2-induced CD3^+^ T cell (**A**), CD3^+^CD4^+^ T cell (**B**), CD4^+^CD154^+^ T cell (**C**), CD3^+^CD8^+^ T cell (**D**), and CD8^+^CD154^+^ T cell (**E**) expression between SARS-CoV-2 seropositive and seronegative individuals among *O. volvulus*-infected individuals following peptide stimulation. Approximately 7 × 10^5^ cells from each donor were stimulated *in vitro* with SARS-CoV-2 peptides (1 µg/mL) for a duration of 6 h. Boxplots represent the median and interquartile range expression of T cells for each group. Comparison was performed using the Mann–Whitney test. “Covid sero +” stands for SARS-CoV-2 seropositive and “Covid sero –” stands for SARS-CoV-2 seronegative. Dots indicate outliers.

**Figure A4:**
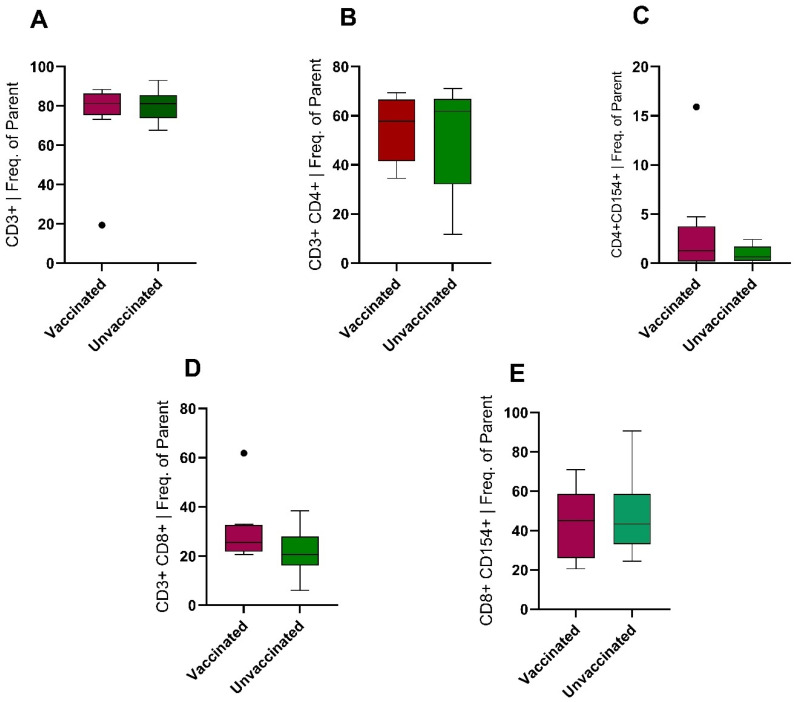
Comparison of SARS-CoV-2-induced CD3^+^ T cell (**A**), CD3^+^CD4^+^ T cell (**B**), CD4^+^CD154^+^ T cell (**C**), CD3^+^CD8^+^ T cell (**D**), and CD8^+^CD154^+^ T cell (**E**) expression between SARS-CoV-2 vaccinated and unvaccinated individuals in *O. volvulus*-infected individuals following peptide stimulation. Approximately 7 × 10^5^ cells from each donor were stimulated *in vitro* with SARS-CoV-2 peptides (1 µg/mL) for a duration of 6 h. Boxplots represent the median and interquartile range expression of T cells for each group. Comparison was performed using the Mann–Whitney test. Dots indicate outliers.

The expression of SARS-CoV-2-induced CD4^+^CD154^+^ and CD8^+^CD154^+^ T cells did not differ significantly between SARS-CoV-2 seropositive and seronegative *O. volvulus*-infected individuals (Figure A3). There was no major difference in SARS-CoV-2-induced T cell activation between vaccinated and unvaccinated *O. volvulus* participants (Figure A4). However, a univariate regression model was used to analyse the relationship between predictor variables, including SARS-CoV-2 IgA, SARS-CoV-2 IgG, NCP IgG, COVID-19 vaccination status, and *A. lumbricoides*-specific IgG levels and SARS-CoV-2-induced CD4^+^CD154^+^ expression as the dependent outcome in *O. volvulus*-infected participants. This analysis revealed a significant association between *A. lumbricoides* IgG and reduced SARS-CoV-2-induced CD4^+^CD154^+^ T cell expression (*β* = −0.499, *p* = 0.025). SARS-CoV-2 IgG was significantly associated with enhanced SARS-CoV-2-induced CD4^+^CD154^+^ (*β* = 0.532, *p* = 0.016). No statistically significant association was found between SARS-CoV-2-induced CD4^+^CD154^+^ expression and other predictors in *O. volvulus*-infected participants, but results suggest a positive association with NCP IgG (*β* = 0.607, *p* = 0.063) (Table 2).

The same model was subsequently applied to assess the relationship between the same predictor variables (SARS-CoV-2 IgA, SARS-CoV-2 IgG, NCP IgG, COVID-19 vaccination status, and *A. lumbricoides*-specific IgG levels) and SARS-CoV-2-induced CD8^+^CD154^+^ expression as the dependent outcome in *O. volvulus*-infected participants (Table 3). SARS-CoV-2 IgG was significantly associated with enhanced SARS-CoV-2-induced CD8^+^CD154^+^ T cells expression (*β* = 0.509, *p* = 0.022) in *O. volvulus*-infected participants, suggesting a positive association. No statistically significant association was observed between *A. lumbricoides* IgG and SARS-CoV-2-induced CD8^+^CD154^+^ T cell expression (*β* = −0.095, *p* = 0.691) (Table 3).

## 4. Discussion

The immune system, ever so complex, offers remarkable insights into how co-infections and prior exposures shape responses to new pathogens. In this study, we probed the impact of helminth infection on viral cellular immunity by stimulating human PBMCs with SARS-CoV-2 peptides and helminth antigens. Using flow cytometry, the expression of SARS-CoV-2-induced CD154 (CD40L) was measured to unravel the immunomodulatory effects of helminths in the context of SARS-CoV-2.

The overall low number of CD154^+^ cells observed in our gating is consistent with the rarity of antigen-specific activated T cells in peripheral blood. In this study, we demonstrated that active helminth infection is associated with reduced cellular immune (CD4^+^CD154^+^) responses to viral infections such as SARS-CoV-2. Individuals with active *O. volvulus* infection exhibited significantly lower expression of SARS-CoV-2–induced CD4^+^CD154^+^ T cells compared to those without filarial worm infection, indicating an immunomodulatory effect in actively infected individuals. Furthermore, we extended these observations by examining the influence of *A. lumbricoides* antigens on SARS-CoV-2-specific T cell responses in *O. volvulus*-infected participants and found that stimulation/exposure to *A. lumbricoides* antigens led to a reduction in SARS-CoV-2-induced CD3^+^ and CD4^+^CD154^+^ T cell expression. Interestingly, we also observed an unexpected increase in CD154 expression on CD8^+^ T cells (CD8^+^CD154^+^) in *O. volvulus*-infected individuals. The active infection in *O. volvulus* participants, coupled with their higher expression of *A. lumbricoides* IgG, is likely to enhance memory T-cell proliferation and a more robust recall response to *A. lumbricoides* antigens during *in vitro* stimulation. This heightened responsiveness may, in turn, enhance regulatory pathways, ultimately resulting in increased suppression of SARS-CoV-2–induced T-cell activity. To our knowledge, this study is among the few to demonstrate the *in vitro* effects of helminth antigens on virus-induced CD4⁺CD154⁺ T-cell responses, highlighting how helminth-driven immune modulation can alter adaptive immune dynamics and potentially influence clinical outcomes of viral infections [21]. Although the mechanisms underlying helminth-virus interactions remain complex and may vary across helminth and viral species, emerging evidence, including our findings, suggests overlapping immunoregulatory pathways, most notably a reduction in Th1-type responses, as reflected by the diminished CD4^+^CD154^+^ T cell activation induced by *A. lumbricoides* and *O. volvulus,* as shown in this study. CD154 (also known as CD40 ligand) is a co-stimulatory molecule primarily expressed on activated CD4^+^ T cells. Its interaction with CD40, which is expressed on antigen-presenting cells, is crucial for initiating and sustaining effective adaptive immune responses [39,40]. Therefore, the reduced expression of CD154 on CD4^+^ T cells in helminth-infected individuals may suggest a reduced ability to provide co-stimulatory help to B cells and dendritic cells [41]. This could lead to suboptimal antibody responses, reduced cytokine production, and a generally dampened adaptive immune response. Suboptimal cytokine and antibody response could also have broad implications for host immune competence, particularly in vaccination contexts which operate through a robust Th1 mechanism. This aligns with existing literature showing that chronic helminth infections can induce a regulatory or Th2-skewed immune environment, promoting immune tolerance and impairing pro-inflammatory responses required to clear pathogens or mount effective vaccine-induced immunity [42,43,44].

The lack of an intrinsic T-cell response to *Ascaris lumbricoides* antigen suggests that it does not act as a strong antigenic stimulus per se. Rather, the observed suppressive effect on SARS-CoV-2-specific responses is most consistent with the induction of a regulatory or immunomodulatory immune environment, mediated by regulatory cell populations and inhibitory cytokines. This phenomenon reflects immune modulation or bystander suppression rather than direct antigen-specific activation and is consistent with observations made using *Litomosoides sigmodontis* antigens, which have been shown to induce TGF-β receptor–responsive, IL-10-producing T cells that suppress bystander T-cell proliferation *in vivo* [45]. This interpretation has implications for understanding COVID-19 immunopathology in helminth-endemic settings. Such helminth-driven immune regulation may contribute to reduced inflammation or cytokine-mediated pathology during SARS-CoV-2 infection, potentially leading to improved clinical outcomes. Conversely, sustained helminth exposure and the associated dampening of CD4^+^CD154^+^ T-helper cell responses may also impair the development of robust antiviral immunity, thereby increasing the risk of suboptimal immune responses upon re-exposure to SARS-CoV-2 [46].

On the other hand, the higher expression of CD154 on CD8^+^ T cells is less well-characterised but has been reported in certain chronic infections and inflammatory conditions [47]. While CD154 expression on CD8^+^ T cells is typically lower than on CD4^+^ T cells, its higher expression may indicate an adaptive or compensatory immune mechanism. This could reflect an attempt by the immune system to maintain some degree of immune activation or cytotoxic function despite the regulatory environment. Alternatively, it might suggest a shift in the role of CD8^+^ T cells, potentially engaging in non-classical helper functions or contributing to immune dysregulation.

The present results align with previous studies that reported helminth-induced inhibition of SARS-CoV-2-reactive CD4^+^ T cells in convalescent COVID-19 patients *in vitro* [31]. However, the frequency of SARS-CoV-2-induced T cell expression observed in the present study was lower, a discrepancy likely attributable to the different origins of the samples used. Unlike the samples from Europe used in previous studies, our samples were derived from individuals in a helminth-endemic region (Africa), where prior helminth exposure is common. Chronic helminth infections are known to induce memory regulatory mechanisms that can dampen immune activation, as previously highlighted [48,49]. This background exposure may result in lower frequencies of activated T cells when stimulated, highlighting the role of geographic and environmental factors in shaping immune responses. Furthermore, the competitive nature of immune responses to different pathogens may make it difficult to tackle helminths and SARS-CoV-2 infection simultaneously with the same magnitude, leading to reduced SARS-CoV-2-specific CD4^+^ T cell response. In addition, chronic infection with *Wuchereria bancrofti* and development of lymphedema have been shown to skew immune priorities driving constant activation and exhaustion of T cells [50,51,52]. These findings illustrate the importance of regional variations in helminth exposure on immune modulation, as chronic helminth endemicity may shape both the magnitude and regulatory capacity of T-cell responses.

The implications of these findings are multifaceted. On one hand, the modulatory effects of helminth antigens could explain the observed variability in disease severity and immune response to SARS-CoV-2 in helminth-endemic regions, potentially offering a degree of protection against hyperinflammation. On the other hand, this reduced T cell expression could hinder the development of effective immune responses, raising concerns about vaccine efficacy and immune memory in infected endemic populations. As the activity of helminths on vaccine efficacy is dependent on the immune target, Th2 responses elicited by helminths may hinder vaccines that target Th1 immune responses, and anthelminthics may be administered first. Moreso, the diminished activation of CD154 on CD4^+^ T cells could impair the establishment of robust antiviral immunity and vaccine efficacy, a hypothesis that merits further exploration, particularly in the context of vaccination strategies in helminth-endemic areas

A drawback to our study is that the uninfected control group did not come from the same region, and stool examinations were not performed to confirm active *A. lumbricoides* infections. Reliance on serology, while valuable for detecting prior exposure, does not clearly distinguish between active/ongoing and past infections, an issue that is particularly relevant in endemic areas where antibody responses are often long-lived and boosted by repeated contact with parasites. Nonetheless, serological assays continue to be indispensable tools for large-scale epidemiological studies, as they reflect immune system reactivity at the population level. Our sample size of 43 participants is relatively small, which may limit the generalisability of our findings. Future studies with larger cohorts will provide more statistical power and strengthen the robustness of the observed effects. While modulation was observed in *A. lumbricoides* and *O. volvulus,* our results cannot fully predict the extent of modulation of other helminths. Future studies incorporating additional helminths, such as hookworm, *S. mansoni*, and *S. stercoralis*, would provide a broader understanding of antigen-specific responses in co-infected individuals. The *in vitro* nature of the experiments, while allowing controlled analysis of specific interactions, may not fully replicate the complex in vivo dynamics between helminths, the immune system, and viral infection. Future studies should aim to incorporate more in vivo models and longitudinal analyses to better understand the long-term effects of helminth-induced immune modulation on virus-specific immunity.

## 5. Conclusions

In conclusion, these findings highlight the possible occurrence of an association between helminths and virus-induced T cell activation, revealing a complex interplay that has critical implications for immune responses in helminth-endemic regions. These insights are particularly relevant for guiding public health strategies, including vaccination strategies, in regions where helminth infections are prevalent.

## Figures and Tables

**Table 1:** Clinical and demographic information of the study participants.

Parameter		LE LF Participants	*O. volvulus*-Infected Participants	Uninfected Control
Gender, n (%)	Male	2 (11.8)	15 (75.0)	4 (66.7)
	Female	15 (88.2)	5 (25.0)	2 (33.3)
Age group, n (%)	17–24	0 (0.0)	2 (10.0)	1 (16.7)
	25–44	7 (41.2)	13 (65.0)	5 (83.3)
	45–64	10 (58.8)	5 (25.0)	0 (0.0)
COVID-19 Sero, n (%)	Seropositive	16 (94.1)	15 (75.0)	6 (100.0)
	Seronegative	1 (5.9)	5 (25.0)	0 (0.0)
Vaccination, n (%)	Vaccinated	7 (41.2)	10 (50.0)	6 (100.0)
	Unvaccinated	10 (58.8)	10 (50.0)	0 (0.0)
Median SARS-CoV-2 IgA titer (ABU)		4 (IQR: 1.6–41.2)	7.9 (IQR: 1.8–16.0)	9.8 (IQR: 4.5–31.1)
Median SARS-CoV-2 IgG titer (ABU)		2 (IQR: 1.2–9.8)	5.4 (IQR: 1.3–8.3)	19.7 (IQR: 6.5–33.2)
Median *A. lumbricoides* IgG titer (Index)		8.3 (IQR: 7.1–10.4)	23.9 (IQR: 16.3–28.5)	9 (IQR: 7.7–10.2)
Lymphedema (LE) staging, n (%)	Stage 1	1 (5.9)	-	-
	Stage 2	11 (64.7)	-	-
	Stage 3	4 (23.5)	-	-
	Stage 6	1 (5.9)	-	-
Average palpable nodules per patient			3	-
Average MF/mg skin snip			56	-
Total investigated, n (%)		17 (100)	20 (100)	6 (100)

This table shows the sociodemographic and clinical characteristics of lymphatic filariasis lymphedema (LF LE), *O. volvulus*-infected participants, and non-endemic controls. LE LF Samples were obtained from participants 36 months after treatment. “COVID-19 Sero” refers to the serological status of individuals with respect to COVID-19 infection. Vaccination refers to COVID-19 vaccination. The different parameters are highlighted in each column with their respective numbers (n) and percentages in brackets. IQR represent the interquartile range. Four (4) individuals were bilaterally affected in the LF LE cohort.

**Table 2:** Summary of univariate regression analysis to explore the relationship between host immune status and SARS-CoV-2-induced CD4^+^CD154^+^ T cell expression in *O. volvulus*-infected participants.

Predictor	*cβ* (Unstandardised)	Std. Error	*β* (Standardised)	t-Value	Sig (*p*-Value)	95% CI for B
SARS IgA	0.075	0.039	0.415	1.937	0.069	[−0.006, 0.156]
SARS IgG	0.190	0.071	0.532	2.662	0.016	[0.040, 0.339]
NCP IgG	3.235	1.496	0.607	2.162	0.063	[−0.215, 6.686]
COVID-19 vaccine	1.989	1.544	0.290	1.288	0.214	[−1.256, 5.234]
*A. lumbricoides* IgG	−0.219	0.09	−0.499	−2.441	0.025	[−0.408, −0.031]

Dependent variable: CD4^+^CD154^+^|Freq. of parent (The frequency of cells was expressed as the percentage of parent cells). N = 20 (*O. volvulus*-infected participants), *β* represents the regression coefficient, and the t-value represents the t-statistic test. Std. Error: represents the standard error.

**Table 3:** Summary of univariate regression analysis to explore the relationship between host immune status and SARS-CoV-2-induced CD8^+^CD154^+^ T cell expression in *O. volvulus*-infected participants.

Predictor	*cβ* (Unstandardised)	Std. Error	*Β* (Standardised)	t-Value	*p*-Value	95% CI for B
SARS IgG	0.981	0.391	0.509	2.511	0.022	[0.160, 1.802]
SARS IgA	0.358	0.213	0.369	1.684	0.109	[−0.089, 0.805]
NCP IgG	8.041	6.163	0.419	1.305	0.228	[−6.170, 22.253]
COVID-19 vaccine	−4.730	8.647	−0.128	−0.547	0.591	[−22.896, 13.436]
*A. lumbricoides* IgG	−0.225	0.558	−0.095	−0.403	0.691	[−1.396, 0.947]

Dependent Variable: CD8^+^CD154^+^|Freq. of Parent (The frequency of cells was expressed as the percentage of parent cells). N = 20 (*O. volvulus*-infected participants), *β* represents the regression coefficient, and the t-value represents the t-statistic test. Std. Error: represents the standard error.

## Data Availability

The data that support the findings of this study are available from the corresponding author upon reasonable request.

## Abbreviations

The following abbreviations are used in this manuscript:

LFLE	Lymphatic Filariasis lymphedema
CFA	Circulating Filarial Antigens
PBMC	Peripheral Blood Mononuclear Cells
FMO	Fluorescence Minus One
SARS-CoV-2	Severe Acute Respiratory Syndrome Coronavirus 2
NCP	Nucleocapsid Protein
SPP	SARS-CoV-2 Peptide Pool
IQR	Interquartile Range
CI	Confidence Interval
KCCR	Kumasi Centre for Collaborative Research in Tropical Medicine
IMMIP	Institute of Medical Microbiology, Immunology and Parasitology
KNUST	Kwame Nkrumah University of Science and Technology
